# Preventive Potential of the Aqueous Extract of the Mixture of *Bidens pilosa* (Asteraceae) and *Cymbopogon citratus* (Poaceae) Aerial Parts on Hypertension Induced by a Chronic Salt and Alcohol Consumption on the Rats

**DOI:** 10.1155/2022/1980622

**Published:** 2022-03-09

**Authors:** Yannick Carlos Tcheutchoua, Danielle Claude Bilanda, Paul Désiré Djomeni Dzeufiet, Oriane Corine Djunie Neali, Pascal Emmanuel Owona, Ronald à Goufani Bidingha, Rodrigue Fifen Ngapout, Lohik Nguegan Mbolang, Michel Noubom, Théophile Dimo, Pierre Kamtchouing

**Affiliations:** ^1^Laboratory of Animal Physiology, Department of Animal Biology, Faculty of Science, University of Yaoundé I, PO Box 812, Yaoundé, Cameroon; ^2^Department of Biological Sciences, Faculty of Medicine, University of Dschang, Po Box 67, Dschang, Cameroon

## Abstract

High blood pressure (HBP) is currently one of the main risk factors for cardiovascular and kidney diseases. Nowadays, populations make extensive use of alternative medicine for their health problems. *Bidens pilosa* (*B*. *pilosa*) and *Cymbopogon citratus* (*C*. *citratus*) are used individually in the traditional treatment of cardiovascular disorders. This study assessed the effects of the mixture of these two plants aqueous extract on HBP in rats. Male rats (42) were divided into 7 groups of 6 rats each. Normotensive rats received only distilled water and formed group 1. The other animals received ethanol + salt preceded by distilled water (10 mL/kg; group 2) and spironolactone (10 mg/kg; group 3); the aqueous extracts of the mixture (100 and 200 mg/kg; groups 4 and 5) isolated plants *B*. *pilosa* (200 mg/kg; group 6) and *C*. *citratus* (200 mg/kg; group 7). Animals were treated for 7 weeks during which water consumption and urine volume were assessed; then, hemodynamic parameters were recorded, and rats were sacrificed. Serum and some organs (liver, kidney, heart, and aorta) were used to evaluate biochemical parameters. Ingestion of ethanol + salt leads to a significant increase in urinary volume and water intake that were significantly prevented by the extracts from the mixture and isolated plants. Ethanol + salt solution significantly increased the blood pressure, heart rate, triglycerides (TG), total cholesterol (TC), low-density lipoprotein cholesterol (LDL-chol), very-low-density lipoprotein cholesterol (VLDL-chol), atherogenic indices, liver and kidney function parameters, and malondialdehyde (MDA) levels. However, the levels of high-density lipoprotein cholesterol (HDL-chol), albumin, reduced glutathione (GSH), catalase, and superoxide dismutase (SOD) activity were significantly reduced. The extracts of the mixture and isolated plants significantly prevented all these variations with a more pronounced action for the lowest dose of the mixture on the lipid profile, oxidative stress, and kidney function. These observations confirm the beneficial effects of *B*. *pilosa* and *C*. *citratus* to manage hypertension.

## 1. Introduction

Arterial hypertension (AHT) is a very common condition today, but for a long time, it was considered a rare and even nonexistent condition in Africa. Today, hypertension is a real public health problem. It refers to a permanent rise in blood pressure in the arteries, which is based on two points: the rise in blood pressure and its persistence [1].

According to the WHO, 1.28 billion people worldwide between the ages of 30 and 79 have hypertension, and most of them (two-thirds) live in low and middle-income countries [2]. The prevalence of hypertension varies across continents and country revenues. High-income countries have the lowest prevalence of hypertension compared to lower-income countries (40% versus 35% on average) [3]. Also, due to weak health systems in these countries, the number of undiagnosed and/or untreated people with hypertension is very high (46%) [2]. In the African continent, the prevalence of hypertension is the highest (27%) compared to the Americas where it is lower (18%) [2]. The prevalence of hypertension in Cameroon was estimated at 29.70% in 2015 [4]. According to a WHO analysis of ten countries on the African continent, cardiovascular disease, diabetes, and asthma are the comorbidities most associated with patients with COVID-19. South Africa, which recorded almost half of the cases and deaths on the continent, found that more than 60% of COVID-19 patients in hospitals had HBP and at least 50% had diabetes [5]. The increase in the prevalence of hypertension is attributable to population growth, ageing populations, and behavioural risk factors such as poor diet, alcohol and salt abuse, lack of physical activity, overweight, and constant exposure to stress [3]. Today, alcoholic beverages are consumed regularly by most societies in the world. However, its abuse is a public health problem [6]. Several studies have shown that chronic heavy alcohol consumption most often causes liver, gastrointestinal, nerve, and cardiovascular damages leading to physiological dysfunction [7, 8]. It has been clearly established that chronic ethanol consumption leads to increased blood pressure and thus hypertension. Global salt consumption is far too high in both developed and developing countries [9]. Many epidemiological studies have established a causal relationship between high dietary sodium consumption and increased blood pressure and thus the risk of hypertension, which in the long term can lead to cardiovascular, liver, and kidney disorders [9]. Chronic consumption of both (alcohol and salt) could have adverse effects on the cardiovascular system, liver, and kidneys.

HBP is a chronic disease that requires lifelong treatment with dietary management and regular use of medications such as ACE inhibitors, calcium channel blockers, and diuretics. These drugs have several limitations, namely, side effects, high cost, and inaccessibility. Despite a large number of modern medicines, people around the world are increasingly turning to herbal medicines for their primary health problems.

African flora in general and Cameroonian flora in particular abounds in diverse varieties of plant species. These plants are most often used by populations for the treatment of several ailments. They are used to treat sexual and fertility disorders, diabetes, obesity, and cardiovascular disorders [10]. Nowadays, the identification of new active ingredients and the discovery of new pharmacological properties, with minimal adverse effects, have contributed to promoting phytotherapy [11]. In view of the important place that these plants occupy in pharmacopoeia, the WHO has recommended that investigations be conducted on the effects of plants used in traditional medicine [12].

Despite the extensive use of herbs in traditional medicine, failures in monotherapy have most often been encountered in the treatment of various diseases. These resistances encountered with monotherapy are leading healthcare personnel to advocate more and more for polytherapy. This renewed interest in the use of several molecules in combination with the treatment of diseases is related to both efficacy and the reduction of doses and possible side effects. Therefore, since several plants have already proven their efficacy and limitations in monotherapy, there is an urgent need to develop new drugs combining several plants for the treatment of diseases such as hypertension.

*B*. *pilosa* is a perennial plant belonging to the Asteraceae family, globally distributed in tropical regions. Scientific studies, although not exhaustive, have shown that *B*. *pilosa* extracts are antitumor, anti-inflammatory, antioxidant, diuretic, antihypertensive, vasorelaxant, and antiulcerative [13–16]. In traditional medicine, the decoction of the whole plant is used against flu and fevers, while the leaves of the plant are used to treat ear infections, psoriasis, hepatitis, and hemorrhoids [15]. The plant is also considered to be antipoisonous [15]. *C*. *citratus* commonly known as lemongrass is an herb belonging to the Poaceae family. Scientific studies have shown that *C*. *citratus* has antispasmodic, antipyretic, sedative, diuretic, vasorelaxant, and hypotensive activities [17–20]. In traditional medicine, *C*. *citratus* is used for its many therapeutic properties. The decoction of the leaves is used to treat gastrointestinal pain, herpes, fever, headaches, and heart problems [19]. The two plants are diuretic, vasorelaxant, and antihypertensive, and so far, no scientific study has been conducted on the effects of the mixture. Therefore, we propose to evaluate the preventive effects of the aqueous extract of the mixture of these two plants on a rat model of hypertension, using alcohol and salt.

## 2. Materials and Methods

### 2.1. Animal Material

Male Wistar rats aged 6–8 weeks and weighing 150–160 g were randomly selected from the colony of Animal Biology and Physiology Animal House of Yaoundé I University. They were reared in Plexiglas cages. Every effort was made to minimise animal suffering and to reduce the number of animals used. The rats were housed 3 per cage and exposed to a daily 12-hour light/dark cycle. They were maintained at room temperature (25 ± 3°C) with free access to a standard diet (composed of 40% maize, 20% wheat bran, 24% fish meal, 4% palm kernel meal, 8% peanut meal, 2% cottonseed meal, 2% bone meal, and a 10 g vitamin complex) and tap water. All procedures and protocols involving animals and their care were conducted in accordance with institutional guidelines and approved by the National Ethics Committee of Cameroon (Reg. No. FWA-IRB00001954).

### 2.2. Plant Material

The aerial parts of *C*. *citratus* (leaves and stems) and *B*. *pilosa* (leaves, stems, and flowers) were collected in Yaoundé (Central Cameroon) more precisely in Messa-ssi in February 2018. *C*. *citratus* was identified by comparison with the botanical collection of D. Dang No. 202 registered at the Yaoundé National Herbarium under No. 18626/SRFcam, and *B*. *pilosa* was identified by comparison with the botanical collection of B. Pollard No. 2 registered at the Yaoundé National Herbarium under No. 60447/HNC.

### 2.3. Plants Extraction

The collected aerial parts of *C*. *citratus* and *B*. *pilosa* were washed, shade-dried, and crushed to obtain the powder of each plant. 200 g of each plant was separately introduced into 2 L of boiling distilled water. The infusion was done until it cooled down. The solutions obtained were filtered with Whatman No. 3 paper, and the filtrates were oven-dried at 45°C to give 10.04 g and 15.96 g of aqueous extract of *B*. *pilosa* and *C*. *citratus*, respectively, for a yield of 5.02% and 7.98%. The doses of the mixture of the two plants were chosen on the basis of screening for direct hypotensive effects. This screening was carried out using combinations of proportions 25–75, 50–50, and 75–25 of the mixture of aqueous extracts of *B*. *pilosa* and *C*. *citratus*. The best combination (50–50) of the mixture was chosen and represented the dose of 100 mg/kg. This dose was multiplied by two to obtain the dose of 200 mg/kg. Knowing the best combination, the doses of 125 g for *B*. *pilosa* and 200 g for *C*. *citratus* were obtained by setting the mass of extract obtained after extraction to 50 g for each plant and also by using the extraction yields of each separate plant. Thus, the amount of crude plant of each plant per 50 g of extract was determined by calculation. 125 g of *B*. *pilosa* and 200 g of *C*. *citratus* were introduced into 3.25 L of boiling distilled water. This gave 33 g of aqueous extract of the mixture of *B*. *pilosa* and *C*. *citratus*, a yield of 10.15%.

### 2.4. Phytochemical Analysis

The secondary metabolites tested were flavonoids, alkaloids, quinones, saponins, steroids, triterpenes, glycosides, anthocyanins, tannins, and phenols.

### 2.5. Screening for Diuretic Activity

All extracts were tested for diuretic activity using the method described by Kau et al. [21]. After checking the urinary excretion, the animals with the best urine volume were selected and divided into 6 groups of 6 animals each. The first group (TN) was treated with distilled water (10 mL/kg) and the second and third (BC 100 and BC 200) with the aqueous extract of the mixture of *B*. *pilosa* and *C*. *citratus* at doses of 100 and 200 mg/kg, respectively. The fourth and fifth groups (B 100 and C 200) were treated with aqueous extracts of *B*. *pilosa* (200 mg/kg) and *C*. *citratus* (200 mg/kg), respectively, while the sixth group (FURO 10) was treated with furosemide (10 mg/kg). The animals of the different groups were placed in standard metabolic cages and food and water were removed 12 hours before the experiment. All extracts were dissolved in distilled water to the required concentrations and administered orally by gavage. Cumulative urine excretion was measured at 3, 6, 9, 12, and 24 hours in all groups. The ratio of urine excretion in the test group to urine excretion in the control group is used as a value of the diuretic action for a given dose of a drug. As diuretic action is subjected to variability, diuretic activity was calculated [21]. To obtain the diuretic activity, the diuretic action of the test substance was compared to that of a standard drug in the test group. The diuretic activity was considered as “no,” “low,” “moderate,” and “good” if the values were, respectively, <0.72, 0.72–1.00, 1.00–1.5, and > 1.5 [21].

Urinary excretion (UE) = total urine output ×100/total fluid administered; diuretic action = urinary excretion of the test group/urinary excretion of the control group; diuretic activity = diuretic action of the test group/diuretic action of a standard drug.

### 2.6. Animal Grouping, Treatment, and Hypertension Induction

The preventive effects of the mixture of *B*. *pilosa* and *C*. *citratus* were evaluated in normotensive male Wistar rats. The plants extracts and the hypertension inducer made up of 40° alcohol (5 g/kg) + salt (2.46 g/kg) for 49 days (7 weeks) were given concomitantly to the animals starting with the extracts. Forty-two (42) male rats were divided into seven groups (1–7) of 6 animals each. These seven groups were treated daily by gavage for 49 days as follows: groups 1 and 2 were receiving distilled water (10 mL/kg), group 3 spironolactone (10 mg/kg), groups 4 and 5 the aqueous extract of the mixture of *B*. *pilosa* and *C*. *citratus* at doses of 100 and 200 mg/kg, respectively, and groups 6 and 7 the aqueous extracts of *B*. *pilosa* (200 mg/kg) and *C*. *citratus* (200 mg/kg), respectively. Thirty minutes after, the inducer (alcohol + salt solution) was administrated to rats in groups 2–7 at a rate of 1.66 mL/100 g bodyweight; while, group 1 was considered as the normotensive control group. Water intake and urinary excretion volume were assessed at the beginning and the end of the experiment using metabolic cages.

### 2.7. Drugs and Chemicals

All the drugs and chemicals used in this study were purchased from Sigma Chemical Company (St. Louis, MO, USA).

### 2.8. Hemodynamic Parameters Recording

At the end of the respective treatments, arterial blood pressure and heart rate of all rats were measured as previously described [22]. Briefly, the rat was anaesthetised by an intraperitoneal injection of urethane (1.5 g/kg). The trachea was exposed and connected to a pipe to allow breathing. The arterial blood pressure was measured from a carotid artery through an arterial pipe connected to a pressure transducer coupled with a hemodynamic recorder Biopac Student Lab. (MP35) and computer.

### 2.9. Blood and Organs Collection

Immediately after hemodynamic parameters measurement, blood samples were collected from the abdominal artery and centrifuged at 3000 rpm for 15 min. The plasma obtained was kept at −20°C for biochemical analysis. Thereafter, the heart, the kidney, the liver, and the thoracic aorta were dissected out, washed in saline, and weighed; parts of all these organs were kept for oxidative stress markers. For histological analysis, other parts of the kidney, the liver, and the aorta were fixed in 10% formalin.

### 2.10. Biochemical Analysis

The heart, aorta, liver, and kidney kept for oxidative stress markers were homogenised in Mc Even solution for the heart and aorta (20% and 10% w/v, respectively) or in Tris-HCl 50 mM buffer solution for the liver and kidney (20%, *w*/v). Tissue protein concentration was assayed [23] using the Biuret reagent. MDA, SOD, catalase, and GSH were determined [24–27]. The concentrations of TC, HDL-chol, TG, creatinine, uric acid, urea, albumin, and bilirubin levels in serum were determined using commercial diagnostic kits (Labkit, ESP). The activities of alanine and aspartate aminotransferases were also determined spectrophotometrically using commercial diagnostic kits (Labkit, ESP). LDL-chol, VLDL-chol, and atherogenic indices were calculated using the method of Ikewuchi [28]: cardiac risk ratio (CRR) = TC/HDL-chol; atherogenic coefficient (AC) = (TC-HDL-chol)/HDL-chol; atherogenic index of plasma (AIP) = log10 (TG/HDL-chol); LDL-chol = (TC–HDL-chol)- (TG/5); and VLDL-chol = TG/5. Creatinine clearance was calculated using the method of Ojeda et al. [29]: creatinine clearance =(*U*× V)/P, where U is the creatinine level in urine, V the volume of urine collected in 24 h, and P the level of creatinine in plasma.

### 2.11. Histological Analysis

For microscopic evaluation, the investigated organs were dehydrated and paraffin-embedded for microscopic examination in accordance with routine laboratory procedures. Paraffin sections of 4 *µ*m were prepared and stained with haematoxylin and eosin (H&E) for histological examination. Morphometric measurements of the thickness of arteries were performed using Image *J* 1.3.

### 2.12. Statistical Analysis

Results were expressed as the mean ± SEM. The difference between the groups was compared using one-way analysis of variance (ANOVA) followed by Turkey's post hoc test using GraphPad Prism software version 8.01. A value of *p* < 0.05 was considered statistically significant.

## 3. Results

### 3.1. Phytochemical Analysis of the Mixture of *B*. *pilosa* and *C*. *citratus* Aerial Parts Aqueous Extract

#### 3.1.1. Qualitative Phytochemical Composition of the Aqueous Extract of the Mixture of *B*. *pilosa* and *C*. *citratus*

The qualitative phytochemical analysis of the aqueous extract of the mixture of *B*. *pilosa* and *C*. *citratus* revealed the following bioactive compounds: flavonoids, alkaloids, quinones, saponins, steroids, triterpenes, glycosides, and phenols. However, anthocyanins and tannins were absent in this extract (Table 1).

#### 3.1.2. Quantitative Phytochemical Composition of the Aqueous Extract of the Mixture of *B*. *pilosa* and *C*. *citratus*

With the quantitative phytochemical analysis of the aqueous extract of the mixture of *B*. *pilosa* and *C*. *citratus*, the polyphenols and flavonoids were quantified (Table 2). The level of polyphenols was 922.71 ± 3.22 mg catechin equivalent per *g* extract, while the level of flavonoids was 71.48 ± 3.41 mg catechin equivalent per *g* extract.

### 3.2. Diuretic Effects of the Aqueous Extract of the Mixture of *B*. *pilosa* and *C*. *citratus*

#### 3.2.1. Effects on Urinary Excretion Volume

**Figure 1:**
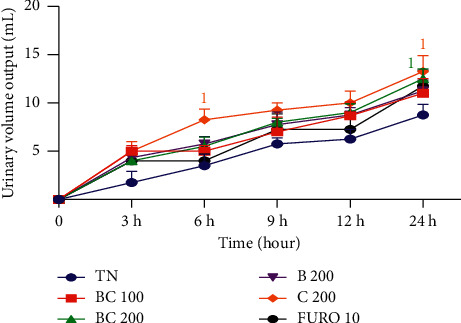
Effects of the aqueous extract of the mixture of *B*. *pilosa* and *C*. *citratus* on urine volume. Each point represents the mean ± SEM (*n* = 6). TN, rats receiving distilled water (10 mL/kg); BC 100 and BC 200, rats receiving the aqueous extract of the mixture of *B*. *pilosa* and *C*. *citratus* at the doses of 100 and 200 mg/kg; FURO 10, rats receiving furosemide at the dose of 10 mg/kg; B 200, rats receiving *B*. *pilosa* aqueous extract at the dose of 200 mg/kg; C 200, rats receiving *C*. *citratus* aqueous extract at the dose of 200 mg/kg; ^1^*p* < 0.05, significant difference compared to the rats receiving distilled water.

The diuretic effects of the aqueous extract of the mixture of *B*. *pilosa* and *C*. *citratus* are shown in Figure 1. 24 hours after administration of the different substances, the aqueous extract of the mixture resulted in a significant (*p* < 0.05) increase in the urine volume at the dose of 200 mg/kg compared to rats given distilled water. *C*. *citratus* aqueous extract (200 mg/kg) caused a significant (*p* < 0.05) increase in urinary volume excreted at 6 and 24 hours compared to rats given distilled water.

#### 3.2.2. Effects on Diuretic Activity

Details of the urinary excretion rate, diuretic action, and diuretic activity are given in Table 3. The results showed that furosemide and extracts increased urine output at 24 h compared to control rats. Like furosemide and separate extracts, the aqueous extract of the mixture of *B*. *pilosa* and *C*. *citratus* showed diuretic activity at all doses. The diuretic activity of a drug is considered to be nil if it is less than 0.72, low if it is between 0.72 and 1.00, moderate if it is between 1.00 and 1.50, and good if it is greater than 1.50. With respect to this, the aqueous extract of the mixture of *B*. *pilosa* and *C*. *citratus* shows a good diuretic activity much more at the dose of 200 mg/kg.

#### 3.2.3. Effects on Water Intake and Urinary Excretion Volume

**Figure 2:**
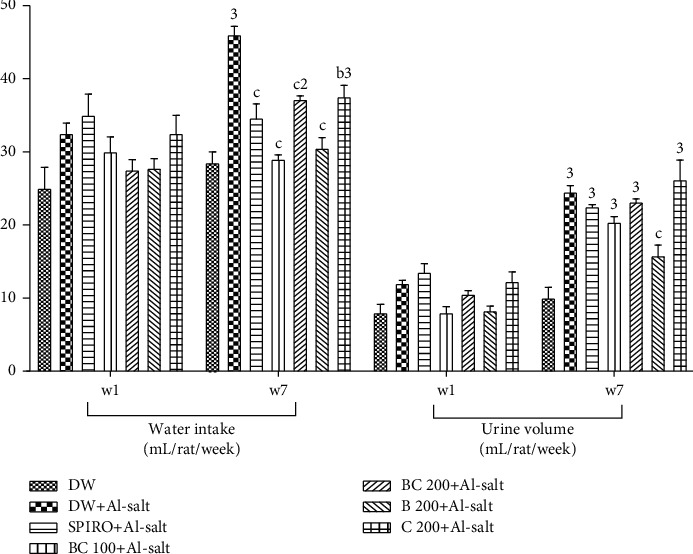
Effects of the aqueous extract of the mixture of *B*. *pilosa* and *C. citratus* on water intake and urinary excretion volume. Each bar represents the mean ± SEM (*n* = 6). DW, normal rats receiving only distilled water (10 mL/kg); DW + Al-salt, rats receiving distilled water and the inducer (alcohol + salt solution) simultaneously; BC 100+Al-salt and BC 200+Al-salt, rats receiving the aqueous extract of the mixture of *B*. *pilosa* and *C*. *citratus* at doses of 100 and 200 mg/kg, respectively, and the inducer simultaneously; SPIRO + Al-salt, rats receiving concomitantly spironolactone at a dose of 10 mg/kg and the inducer; B 200+Al-salt, rats receiving concomitantly *B*. *pilosa* aqueous extract (200 mg/kg) and the inducer; C 200+Al-salt: rats receiving *C*. *citratus* aqueous extract (200 mg/kg) and the inducer simultaneously; ^2^*p* < 0.01; ^3^*p* < 0.001, significant difference compared to normal rats receiving only distilled, ^b^*p* < 0.01; ^c^*p* < 0.001, significant difference compared to rats receiving distilled water and the inducer.

Administration of distilled water (10 mL/kg) concomitantly with the inducer (ethanol + salt solution, hypertensive rats) induced a significant (*p* < 0.001) increase in water intake and urinary excretion in rats compared to rats given distilled water alone (normotensive rats, Figure 2). The increase in water intake induced by alcohol + salt solution was significantly (*p* < 0.001) prevented by the aqueous extract of *B*. *pilosa* and *C*. *citratus* mixture (100 and 200 mg/kg), *B*. *pilosa* (200 mg/kg), and spironolactone (10 mg/kg) by 24.72%, 36.95%, 19.21%, and 33.69%, respectively, compared to hypertensive rats. This increase induced by alcohol + salt solution was also significantly (*p* < 0.01) prevented in animals treated concomitantly with *C*. *citratus* (200 mg/kg) and the inducer by 18.47%. At the end of the experiment, a significant (*p* < 0.001) decrease in the volume of urinary excretion was observed in rats treated concomitantly with inducer and *B*. *pilosa* aqueous extract (200 mg/kg) by 35.71% as compared to hypertensive rats.

### 3.3. Effects of the Aqueous Extract of the Mixture of *B*. *pilosa* and *C*. *citratus* on Blood Pressure and Heart Rate

**Figure 3:**
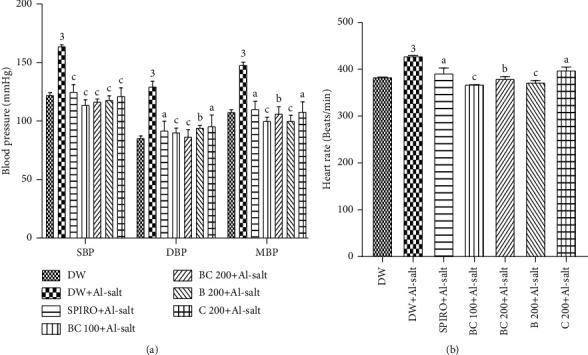
Effects of the aqueous extract of the mixture of *B*. *pilosa* and *C*. *citratus* on blood pressure (a) and heart rate (b). Each bar represents the mean ± SEM (*n* = 6). SBP, systolic blood pressure; DBP, diastolic blood pressure; MBP, mean blood pressure; DW, normal rats receiving only distilled water (10 mL/kg); DW + Al-salt, rats receiving distilled water (10 mL/kg) and the inducer (alcohol + salt solution) concomitantly; BC 100+Al-salt and BC 200+Al-salt, rats receiving the aqueous extract of *B*. *pilosa* and *C*. *citratus* mixture at 100 and 200 mg/kg, respectively, and the inducer concomitantly; SPIRO + Al-salt, rats receiving spironolactone (10 mg/kg) and the inducer simultaneously; B 200+Al-salt, rats receiving *B*. *pilosa* aqueous extract (200 mg/kg) and the inducer simultaneously; C 200+Al-salt, rats receiving *C*. *citratus* extract (200 mg/kg) and the inducer; ^3^*p* < 0.001, significant difference compared to normotensive rats receiving only distilled water; ^a^*p* < 0.05; ^b^*p* < 0.05; ^c^*p* < 0.001, significant difference compared to rats given distilled water and the inducer.

Administration of the inducer (ethanol + salt solution) and distilled water (10 mL/kg) to normotensive rats for 7 weeks resulted in a significant (*p* < 0.001) increase in systolic (SBP), diastolic (DBP), and mean (MBP) blood pressure and heart rate (Figure 3). This increase was 34.09%, 51.37%, 37.42%, and 15.41%, respectively, compared to normotensive rats. The increase in SBP, DBP, MBP, and heart rate observed in the group treated with distilled water and the inducer (hypertensive rats) was significantly (*p* < 0.001) prevented in the group treated concomitantly with aqueous extract of *B*. *pilosa* and *C*. *citratus* mixture (100 mg/kg) and the inducer by 30.51%, 30.16%, 32.30%, and 14.12%, respectively. Treatment concomitantly with the aqueous extract of *B*. *pilosa* and *C*. *citratus* mixture (200 mg/kg) and the inducer significantly prevented the increase in SBP, DBP, MBP, and heart rate. The values decreased by 28.89% (*p* < 0.001), 33.13% (*p* < 0.001), 32.11% (*p* < 0.001), and 11.25% (*p* < 0.01), respectively, compared to hypertensive rats. In animals treated concomitantly with spironolactone and the inducer, there was a significant decrease in SBP, DBP, MBP, and heart rate of 23.67% (*p* < 0.001), 29.06% (*p* < 0.01), 25.45% (*p* < 0.001), and 8.59% (*p* < 0.05), respectively, as compared to hypertensive rats. In animals treated concomitantly with aqueous extract of *B*. *pilosa* (200 mg/kg) or *C*. *citratus* (200 mg/kg) and the inducer, there was a significant decrease in SBP (*p* < 0.001), DBP (*p* < 0.01; *p* < 0.005, respectively), MBP (*p* < 0.001; *p* < 0.005, respectively), and heart rate (*p* < 0.001; *p* < 0.05, respectively) compared to hypertensive rats.

### 3.4. Effects of the Aqueous Extract of the Mixture of *B*. *pilosa* and *C*. *citratus* on the Lipid Profile

Administration of the inducer (ethanol + salt solution) and distilled water (10 mL/kg) to normotensive rats resulted in a significant (*p* < 0.001) increase in TC by 42.40%, TG by 117.23%, LDL-chol by 863.03%, and VLDL-chol by 117.23% (Table 4) as compared to normal rats receiving only distilled water (normotensive rats). The increase in TG and VLDL-chol induced by ethanol + salt solution was significantly (*p* < 0.001) prevented by the aqueous extract of the mixture of *B*. *pilosa* and *C*. *citratus* (100 mg/kg) by 62.31% and 62.33%, respectively, as compared with hypertensive rats. The mixture's aqueous extract (200 mg/kg) significantly (*p* < 0.01) prevented the increase induced by the alcohol + salt solution in TG and VLDL-chol by 33.48% and 33.53%, respectively, as compared to hypertensive rats. The mixture's aqueous extract at doses of 100 mg/kg (*p* < 0.001) and 200 mg/kg (*p* < 0.05) significantly prevented the increase in TC induced by the alcohol + salt solution as compared with the hypertensive rats. The aqueous extract of the mixture of *B*. *pilosa* and *C*. *citratus* (200 mg/kg) significantly (*p* < 0.01) prevented the decrease induced by the alcohol + salt solution in HDL-chol by 33.21% as compared with the hypertensive rats. In animals given alcohol + salt and spironolactone (10 mg/kg), there was a significant (*p* < 0.01) prevention of the increase induced by the alcohol + salt solution in TC levels by 12.34%, in TG (*p* < 0.001) by 53.38%, in LDL-chol (*p* < 0.05) by 41.42%, and in VLDL-chol (*p* < 0.001) by 62.33% as compared to hypertensive rats. *B*. *pilosa* aqueous extract (200 mg/kg) significantly prevented the increase induced by the alcohol + salt solution in TG levels (*p* < 0.001) by 60.79% and in VLDL-chol (*p* < 0.001) by 60.76% as compared to hypertensive rats. The aqueous extract of *C*. *citratus* (200 mg/kg) significantly prevented the increase induced by the alcohol + salt solution in TG (*p* < 0.001) by 53.81%, in LDL-chol (*p* < 0.01) by 55.67%, and in VLDL-chol (*p* < 0.001) by 53.81% and the decrease in HDL-chol by 50.43% (*p* < 0.001) as compared with hypertensive rats.

### 3.5. Effects of the Aqueous Extract of the Mixture of *B*. *pilosa* and *C*. *citratus* on Atherogenic Indices

The effects of the aqueous extract of the mixture of *B*. *pilosa* and *C*. *citratus* on the levels of the atherogenic indices are given in Table 5. Administration of the inducer (alcohol + salt solution) and distilled water (10 mL/kg) resulted in a significant (*p* < 0.001) increase in CRR, AC, and AIP compared to normal rats given distilled water alone (normotensive rats). The aqueous extract of the mixture of *B*. *pilosa* and *C*. *citratus* (100 mg/kg) significantly prevented the increase induced by the alcohol + salt solution in CRR (*p* < 0.001), AC (*p* < 0.01), and AIP (*p* < 0.001) as compared with hypertensive rats. The aqueous extract of the plant mixture (200 mg/kg) significantly (*p* < 0.001) prevented the increase induced by the alcohol + salt solution in CRR, AC, and AIP as compared with hypertensive rats. Treatment concomitantly with alcohol + salt and *C*. *citratus* aqueous extract (200 mg/kg) significantly (*p* < 0.001) prevented increases induced by the alcohol + salt solution in CRR, AC, and AIP as compared with hypertensive rats. The aqueous extract of *B*. *pilosa* aqueous extract (200 mg/kg) significantly (*p* < 0.001) prevented the increase induced by the alcohol + salt solution in AIP as compared with hypertensive rats. Treatment concomitantly with alcohol + salt and spironolactone (10 mg/kg) significantly prevented the increase of CRR (*p* < 0.001), AC (*p* < 0.05), and AIP (*p* < 0.001) as compared with hypertensive rats.

### 3.6. Effects of the Aqueous Extract of the Mixture of *B. pilosa* and *C*. *citratus* on Some Markers of Liver Function

Table 6 provides the preventive effects of the aqueous extract of the mixture of *B*. *pilosa* and *C*. *citratus* on some liver function markers. Administration of the inducer (alcohol + salt solution) and distilled water (10 mL/kg) resulted in a significant increase in the activities of aspartate aminotransferase (AST) by 645.95% (*p* < 0.001), alanine aminotransferase (ALT) by 40.41% (*p* < 0.01), and bilirubin concentration by 33.33% (*p* < 0.01) and a significant decrease in albumin levels by 24.79% (*p* < 0.01) compared to normal rats receiving distilled water only (normotensive rats). The aqueous extract of the mixture of *B*. *pilosa* and *C*. *citratus* at doses of 100 and 200 mg/kg or spironolactone (10 mg/kg) significantly (*p* < 0.001) prevented the increase induced by the alcohol + salt solution in ALT activity by 55.80%, 76.43%, and 58.34%, respectively, as compared with hypertensive rats. These solutions had the same effect on AST activity by 54.70% (*p* < 0.001), 15.36% (*p* < 0.05), and 28.53% (*p* < 0.001), respectively, as compared with hypertensive rats. The aqueous extract of the mixture of *B*. *pilosa* and *C*. *citratus* (100 and 200 mg/kg) and spironolactone also significantly prevented the increase induced by the alcohol + salt solution in total bilirubin by 45.83% (*p* < 0.001), 25.00% (*p* < 0.01), and 20.83% (*p* < 0.05), respectively, as compared with hypertensive rats. In rats treated simultaneously with alcohol + salt solution and the aqueous extract of the mixture of *B*. *pilosa* and *C*. *citratus* (200 mg/kg), there was a significant difference in ALT activity of 75.80% (*p* < 0.001) compared to normotensive rats. In rats treated simultaneously with alcohol + salt solution and *C*. *citratus* (200 mg/kg), there was a significant prevention of increase in ALT activity (*p* < 0.001), AST (*p* < 0.001) and total bilirubin level (*p* < 0.01) as compared with hypertensive rats. The aqueous extract of *B*. *pilosa* (200 mg/kg) significantly prevented the increase induced by alcohol + salt solution in ALT (*p* < 0.001) and AST (*p* < 0.001) activities and total bilirubin level (*p* < 0.05) as compared with hypertensive rats.

### 3.7. Effects of the Aqueous Extract of the Mixture of *B. pilosa* and *C. citratus* on Some Markers of Renal Function

#### 3.7.1. In the Serum

Table 7 provides the preventive effects of the aqueous extract of the mixture of *B*. *pilosa* and *C*. *citratus* on some markers of renal function at the serum level. A significant increase in serum creatinine by 37.66% (*p* < 0.001), serum uraemia by 183.40% (*p* < 0.001), and serum uric acid by 202.28% (*p* < 0.001) was observed in rats receiving distilled water (10 mL/kg) and the inducer (alcohol + salt solution) concomitantly, compared with normotensive rats. In rats given inducer and distilled water (hypertensive rats), a decrease in creatinine clearance of 50.00% (*p* < 0.01) was observed as compared to normotensive rats. The aqueous extract of the mixture of *B*. *pilosa* and *C*. *citratus* (100 and 200 mg/kg) and spironolactone significantly prevented increases induced by the alcohol + salt solution in creatinine levels, respectively, by 34.90% (*p* < 0.001), 18.86% (*p* < 0.05), and 30.18% (*p* < 0.001), urea levels, respectively, by 85.26% (*p* < 0.001), 69.92% (*p* < 0.001), and 66.09% (*p* < 0.001), and uric acid levels, respectively, by 81.25% (*p* < 0.001), 63.79% (*p* < 0.001), and 64.76% (*p* < 0.001) as compared with hypertensive rats. The aqueous extract of the *B*. *pilosa* and *C*. *citratus* mixture (100 mg/kg) possesses significant (*p* < 0.001) increase in creatinine clearance of 138.89% as compared with hypertensive rats. In rats treated concomitantly with alcohol + salt and the mixture's aqueous extract (200 mg/kg), there was a significant difference in creatinine of 11.68% (*p* < 0.01) as compared to normotensive rats. In rats treated concomitantly with alcohol + salt solution and *C*. *citratus* aqueous extract (200 mg/kg), we observed a significant prevention of the increase induced by the alcohol + salt solution in creatinine (*p* < 0.001), uraemia (*p* < 0.001), and serum uric acid (*p* < 0.001) as compared with hypertensive rats. The aqueous extract of *B*. *pilosa* (200 mg/kg), significantly (*p* < 0.001) prevented the increase induced by the alcohol + salt solution in serum creatinine, uraemia and serum uric acid as compared with hypertensive rats. The aqueous extract of *B*. *pilosa* (200 mg/kg) significantly (*p* < 0.01) prevented the decrease induced by the alcohol + salt solution in creatinine clearance as compared with hypertensive rats.

The alcohol + salt solution resulted in a significant (*p* < 0.01) decrease in magnesium, sodium, and potassium levels of 39.39%, 18.11%, and 46.43%, respectively, as compared to normotensive rats. Treatment concomitantly with inducer and the aqueous extract of the mixture of *B*. *pilosa* and *C*. *citratus* (100 mg/kg) or *B*. *pilosa* aqueous extract (200 mg/kg) significantly (*p* < 0.01) prevented the decrease induced by the alcohol + salt solution in magnesium levels of 49.09% and 52.27%, respectively, as compared to hypertensive rats. The aqueous extract of the mixture of *B*. *pilosa* and *C*. *citratus* at doses of 100 and 200 mg/kg significantly (*p* < 0.01) prevented the decrease induced by the alcohol + salt solution in sodium levels by 26.39% and 15.62%, respectively, as compared with hypertensive rats. The aqueous extract of *C*. *citratus* (200 mg/kg) and spironolactone (10 mg/kg) significantly (*p* < 0.001) prevented the decrease induced by the alcohol + salt solution in sodium levels by 28.33% and 21.16%, respectively, as compared to hypertensive rats.

#### 3.7.2. Urinary Level Effects

At the urinary level, the alcohol + salt solution resulted in a significant (*p* < 0.001) decrease in creatinine, urea, and uric acid levels of 50.88%, 79.67%, and 55.72%, respectively, as well as a significant increase (*p* < 0.001) in magnesium, sodium, and potassium levels of 158.15%, 33.07%, and 181.78%, respectively, as compared to normotensive rats (Table 8). The aqueous extract of the mixture of the plants (100 and 200 mg/kg) significantly prevented the decrease induced by inducer in creatinine (*p* < 0.001; *p* < 0.01, respectively) and urea (*p* < 0.001; *p* < 0.05, respectively) levels as well as the increase (*p* < 0.001) of sodium and potassium levels as compared with hypertensive rats. Treatment concomitantly with alcohol + salt solution and *B*. *pilosa* aqueous extract (200 mg/kg) significantly (*p* < 0.01) prevented the decrease induced by inducer in urea levels and the increase (*p* < 0.001) in sodium and potassium levels compared with hypertensive rats. The aqueous extract of *C*. *citratus* (200 mg/kg) significantly (*p* < 0.05) prevented the decrease induced by inducer in urea and uric acid levels and the increase (*p* < 0.001) in sodium and potassium levels as compared with hypertensive rats. Spironolactone significantly (*p* < 0.001) prevented the decrease in uric acid levels and the increase (*p*< 0.001) in sodium and potassium levels as compared with hypertensive rats.

### 3.8. Effects of the Aqueous Extract of the Mixture of *B*. *pilosa* and *C*. *citratus* on Some Oxidative Stress Parameters

**Figure 4:**
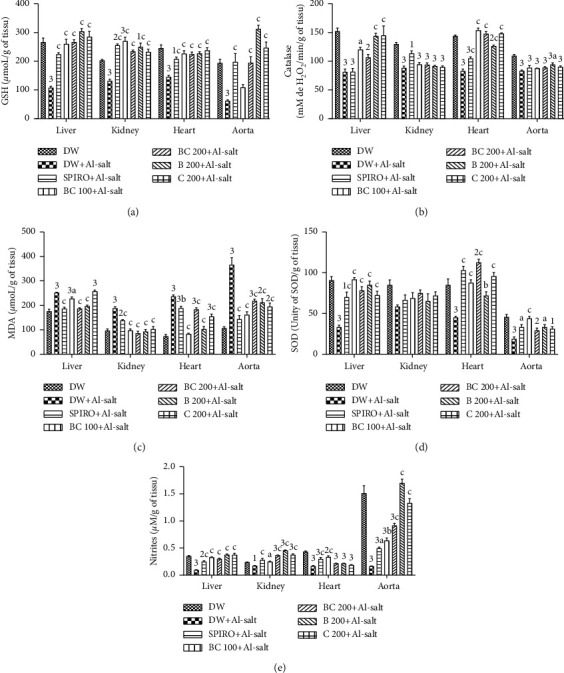
Effects of the aqueous extract of the mixture of *B*. *pilosa* and *C*. *citratus* on the level of GSH, catalase, and SOD activity, on the concentration of MDA and on the level of nitrites. Each bar represents the mean ± SEM (*n* = 6). DW, normal rats receiving only distilled water (10 mL/kg); DW + Al-salt, rats receiving distilled water (10 mL/kg) and the inducer (alcohol + salt solution) simultaneously; BC 100+Al-salt and BC 200+Al-salt, rats receiving the aqueous extract of the mixture of *B*. *pilosa* and *C*. *citratus*, respectively, at doses of 100 and 200 mg/kg and the inducer simultaneously; SPIRO + Al-salt, rats receiving spironolactone (10 mg/kg) and the inducer simultaneously; B 200+Al-salt, rats receiving the aqueous extract of *B*. *pilosa* (200 mg/kg) and the inducer simultaneously; C 200 + Al-salt, rats receiving concurrent the aqueous extract of *C*. *citratus* (200 mg/kg) and the inducer. ^1^*p* < 0.05; ^2^*p* < 0.01; ^3^*p* < 0.001, significant difference compared to normal rats treated with only distilled water; ^a^*p* < 0.05; ^b^*p* < 0.01; ^c^*p* < 0.001, significant difference compared to rats receiving distilled water and the inducer.

Administration of the inducer (ethanol + salt solution) and distilled water (10 mL/kg) to normotensive rats resulted in a significant (*p* < 0.001) decrease in the concentration of GSH in the aorta by 66.06%, the heart by 40.84%, the kidney by 30.62%, and the liver by 59.62% as compared to normotensive rats (Figure 4(a)). The aqueous extract of the mixture of *B*. *pilosa* and *C*. *citratus* (100 mg/kg) prevented this decrease in GSH significantly (*p* < 0.001) in the heart, kidney, and liver. The values were 55.90%, 92.08%, and 142.16%, respectively, higher in these organs as compared to hypertensive rats. The extract of the mixture of *B*. *pilosa* and *C*. *citratus* (200 mg/kg) also significantly (*p* < 0.001) prevented the decrease of GSH induced by alcohol + salt solution in the heart, aorta, kidney, and liver. Values were 56.36%, 171.86%, 66.96%, and 147.96%, respectively, greater in these organs as compared to hypertensive rats. Spironolactone, the aqueous extract of *B*. *pilosa* (200 mg/kg) and *C*. *citratus* (200 mg/kg) significantly (*p* < 0.001) prevented the decrease in GSH levels induced by alcohol + salt solution in all organs as compared to rats given distilled water and the inducer (hypertensive rats).

Catalase activity in the investigated organs of rats concomitantly administered distilled water (10 mg/kg) and the inducer (ethanol + salt solution) was significantly (*p* ˂ 0.001) decreased in the aorta by 24.14%, the heart by 42.58%, the kidney by 31.94%, and the liver by 46.49%, compared to normal rats administered distilled water alone (normotensive rats, Figure 4(b)). This decrease was significantly prevented in the liver by the aqueous extracts of the mixture of *B*. *pilosa* and *C*. *citratus* (100 mg/kg; *p* ˂0.05), *B*. *pilosa* (200 mg/kg; *p* ˂ 0.001), or *C*. *citratus* (200 mg/kg; *p* ˂ 0.001). The values were 47.66%, 76.99%, and 77.67%, respectively, higher as compared to hypertensive rats. The different aqueous extracts and spironolactone significantly (*p* ˂ 0.001) prevented the decrease in catalase activity at the cardiac level as compared to hypertensive rats. At the aortic level, there was a significant (*p* ˂ 0.05) prevention of the decrease in catalase activity induced by alcohol + salt solution in rats, with *B*. *pilosa* extract (200 mg/kg) by 13.23% as compared to hypertensive rats.

Figure 4(c) shows the effects of the aqueous extract of the mixture of *B*. *pilosa* and *C*. *citratus* on MDA levels. It can be seen that the concentration of MDA was significantly increased (*p* < 0.001) in the heart by 263.41%, in the aorta by 241.71%, in the liver by 42.88%, and in the kidneys by 94.48% in rats receiving distilled water and the inducer (hypertensive rats) concomitantly, compared to normotensive rats. The aqueous extract of the mixture of *B*. *pilosa* and *C*. *citratus* significantly prevented the increase (*p* < 0.001) in MDA levels induced by alcohol + salt solution in the aorta, heart, and kidney by 55.95%, by 64.89%, and by 48.04% at 100 mg/kg and by 40.30%, by 22.60%, and by 54.23% at 200 mg/kg, respectively, lower as compared to hypertensive rats. In the liver, the aqueous extract of the mixture of *B*. *pilosa* and *C*. *citratus* (100 and 200 mg/kg) prevented the significant increase in MDA level by 9.68% (*p* < 0.05) and 25.76% (*p* < 0.001), respectively, as compared to hypertensive rats. Treatment concomitantly with alcohol + salt and *B*. *pilosa* aqueous extract (200 mg/kg) significantly (*p* < 0.001) prevented the increase in MDA levels by 56.92% in the heart, 42.17% in the aorta, 50.83% in the kidney, and 21.99% in the liver compared to rats treated with distilled water and inducer. *C*. *citratus* extract (200 mg/kg) produced the same effects in the heart by 34.63%, in the aorta by 46.72%, and in the kidney by 45.12% as compared to hypertensive rats. Spironolactone (10 mg/kg) significantly prevented the increase in MDA levels induced by alcohol + salt solution in the heart by 20.30% (*p* < 0.01), in the liver by 25.75% (*p* < 0.001), in the aorta by 60.75% (*p* < 0.001), and in the kidney by 26.49% (*p* < 0.001) as compared to hypertensive rats.

SOD activity was significantly (*p* < 0.001) decreased in the liver by 63.22%, in the heart by 59.56%, and in the aorta by 91.66% of hypertensive rats as compared to normotensive rats (Figure 4(d)). The aqueous extract of the mixture of *B*. *pilosa* and *C*. *citratus* (100 mg/kg) significantly (*p* < 0.001) prevented the decrease in SOD activity induced by alcohol + salt solution in the liver, heart, and aorta. Values were 175.00%, 154.58%, and 140.38%, respectively, greater in these organs compared to hypertensive rats. The aqueous extract of the mixture of *B*. *pilosa* and *C*. *citratus* (200 mg/kg) also significantly (*p* < 0.001) prevented the decrease in SOD activity induced by alcohol + salt solution in the liver and the heart. The values were 134.38% and 227.30%, respectively, higher in these organs compared to hypertensive rats. At the same dose of the mixture's aqueous extract, SOD activity was significantly (*p* < 0.01) higher in the heart by 32.35% as compared to normotensive rats. Spironolactone significantly prevented the decrease in SOD activity induced by alcohol + salt solution in the liver (*p* < 0.001), heart (*p* < 0.001), and aorta (*p* < 0.05). The values were 110.02%, 200.02%, and 541.02% higher, respectively, in these organs as compared to hypertensive rats. *B*. *pilosa* aqueous extract (200 mg/kg) significantly prevented the decrease in SOD induced by alcohol + salt solution in the liver (*p* < 0.001), heart (*p* < 0.01), and aorta (*p* < 0.05). The values were 155.02%, 109.10%, and 567.62%, respectively, greater in these organs compared to hypertensive rats. The aqueous extract of *C*. *citratus* (200 mg/kg) had the same effect in the aorta by 140.38% (*p* < 0.001) and in the heart by 178.81% (*p* < 0.001) compared to hypertensive rats.

Administration of the inducer (ethanol + salt solution) resulted in a significant (*p* < 0.001) decrease in nitrite levels in the liver by 70.68%, heart by 61.66%, aorta by 86.69%, and kidney by 17.77% (*p* < 0.05) as compared to normotensive rats (Figure 4(e)). The aqueous extract of the *B*. *pilosa* and *C*. *citratus* mixture significantly (*p* < 0.001) prevented the decrease in nitrite levels induced by alcohol + salt solution in the aorta, liver, kidney, and heart. The values were, respectively, by 215.00%, 220.00%, 111.11%, and 106.25% at 100 mg/kg and 355.00%, 200.00%, and 127.77% at 200 mg/kg, higher as compared to hypertensive rats. In the spironolactone (10 mg/kg) treated group, there was a significant (*p* < 0.001; *p* < 0.05 for aorta) prevention of nitrite decline in the heart, liver, kidney, and aorta. Values were 78.91%, 150.00%, 50.00%, and 115.00%, respectively, greater in these organs as compared to hypertensive rats. *B*. *pilosa* aqueous extract (200 mg/kg) significantly (*p* < 0.001) prevented the decrease in nitrite levels in the kidney, liver, and aorta induced by alcohol + salt solution. The values were 114.78%, 266.10%, and 576.78%, respectively, higher in these organs as compared to hypertensive rats. In concomitant treatment with alcohol + salt solution, *C*. *citratus* aqueous extract (200 mg/kg) also significantly (*p* < 0.001) prevented the decrease in nitrite levels in the kidney, liver, and aorta. The values were 74.30%, 265.19%, and 696.48%, respectively, higher in these organs as compared to hypertensive rats.

### 3.9. Effects of the Aqueous Extract of the Mixture of *B*. *pilosa* and *C*. *citratus* on Tissue Protein Levels

**Figure 5:**
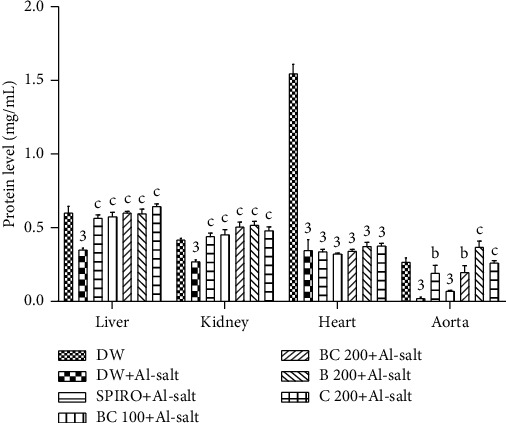
Effects of the aqueous extract of the mixture of *B*. *pilosa* and *C*. *citratus* on tissue protein levels. Each bar represents the mean ± SEM (*n* = 6). DW, normal rats receiving only distilled water (10 mL/kg); DW + Al-salt, rats receiving distilled water (10 mL/kg) and the inducer (alcohol + salt solution) simultaneously; BC 100+Al-salt and BC 200+Al-salt, rats receiving the aqueous extract of the mixture of *B*. *pilosa* and *C*. *citratus*, respectively, at doses of 100 and 200 mg/kg and the inducer simultaneously; SPIRO + Al-salt, rats receiving spironolactone (10 mg/kg) and the inducer simultaneously; B 200+Al-salt, rats receiving *B*. *pilosa* aqueous extract (200 mg/kg) and the inducer simultaneously; C 200 + Al-salt, rats receiving *C*. *citratus* aqueous extract (200 mg/kg) and the inducer simultaneously; ^1^*p* < 0.05; ^3^*p* < 0.001, significant difference compared to normal rats treated with distilled water only; ^a^*p* < 0.05; ^c^*p* < 0.001, significant difference compared to rats receiving distilled water and the inducer solution.

Figure 5 shows the effects of the aqueous extract of the mixture of *B*. *pilosa* and *C*. *citratus* on tissue protein levels. It can be seen that protein levels were significantly (*p* < 0.001) decreased in the aorta, heart, liver, and kidney by 90.85%, 76.02%, 41.66%, and 34.86%, respectively, in hypertensive rats as compared to normotensive rats. The aqueous extract of the plant mixture (100 mg/kg) significantly (*p* < 0.001) prevented the decrease induced by alcohol + salt solution in protein levels in the liver by 64.28% and in the kidney by 66.98%, respectively, as compared to hypertensive rats. The aqueous extract of the mixture of *B*. *pilosa* and *C*. *citratus* (200 mg/kg) significantly prevented the decrease induced by alcohol + salt solution in tissue protein levels in the kidney, liver, and aorta as compared to hypertensive rats. The values were 85.66% (*p* < 0.001), 71.42% (*p* < 0.001), and 712.5% (*p* < 0.01), respectively, higher in these organs as compared to hypertensive rats. Spironolactone (10 mg/kg) administered under the same conditions as the aqueous extract of the plant mixture significantly prevented the decrease in protein levels in the kidney, liver, and aorta. Values were 61.88% (*p* < 0.001), 37.36% (*p* < 0.001), and 691.66% (*p* < 0.01), respectively, greater in these organs as compared to hypertensive rats. The aqueous extracts of *B*. *pilosa* (200 mg/kg) and *C*. *citratus* (200 mg/kg) significantly (*p* < 0.001) prevented the decrease induced by alcohol + salt solution in protein levels in the kidney, liver, and aorta compared to hypertensive rats.

### 3.10. Effects of the Aqueous Extract of the Mixture of *B*. *pilosa* and *C*. *citratus* on Histomorphometry of the Aorta and the Microarchitecture of the Kidney and Liver

**Figure 6:**
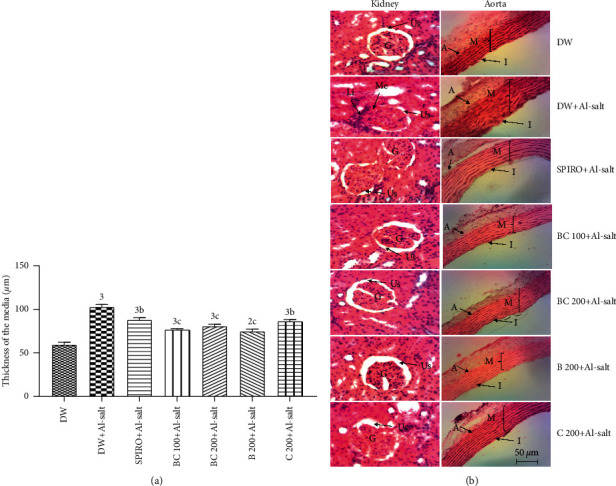
Preventive effects of the aqueous extract of the mixture of *B*. *pilosa* and *C*. *citratus* on histomorphometry (a) of the aorta and the microarchitecture (b) of the kidney (200X, HE) and aorta (100X, HE). Each bar represents the mean ± SEM (*n* = 6). DW, normal rats receiving only distilled water (10 mL/kg); DW + Al-salt, rats receiving distilled water (10 mL/kg) and the inducer (alcohol + salt solution) simultaneously; BC 100 + Al-salt and BC 200 + Al-salt, rats receiving the aqueous extract of the mixture of *B*. *pilosa* and *C. citratus*, respectively, at doses of 100 and 200 mg/kg and the inducer simultaneously; SPIRO + Al-salt, rats receiving concomitantly spironolactone at a dose of 10 mg/kg and the inducer; B 200 + Al-salt, rats receiving concomitantly *B*. *pilosa* aqueous extract (200 mg/kg) and the inducer; C 200+Al-salt, rats receiving concurrent *C*. *citratus* aqueous extract (200 mg/kg) and the inducer. Kidney: *G*, glomerulus; Li, leukocyte infiltration; Us, urinary space; Me, mesangial expansion. Aorta: A, adventitia; *M*, media; *I*, intima.

Figure 6 shows the effects of preventive treatment of the aqueous extract of the mixture of *B*. *pilosa* and *C*. *citratus* on histomorphometry of the aorta (A) and the microarchitecture of the kidney and aorta (B). The administration of the inducer (alcohol + salt solution) in rats resulted in a significant increase in media thickness by 73.84% (*p* < 0.001) as compared to normotensive rats. The aqueous extract of the mixture of *B*. *pilosa* and *C*. *citratus* (100 and 200 mg/kg) significantly (*p* < 0.001) prevented the increase induced by alcohol + salt solution in the thickness of this tunic as compared to hypertensive rats. The values were, respectively, 25.55% and 22.24% lower as compared to hypertensive rats. The aqueous extracts of *B*. *pilosa* (200 mg/kg) and *C*. *citratus* (200 mg/kg) significantly prevented the increase induced by alcohol + salt solution in media size as compared to hypertensive rats. The values were 14.70% (*p* < 0.01), 27.42% (*p* < 0.001), and 16.17% (*p* < 0.01), respectively, lower as compared to hypertensive rats. Treatment with alcohol + salt solution resulted in renal leukocyte infiltration and renal mesenchymal expansion as compared to normotensive rats. The aqueous extract of the mixture of *B*. *pilosa* and *C*. *citratus* as well as the separate extracts of these two plants prevented the damages induced by alcohol + salt solution as compared to hypertensive rats.

## 4. Discussion

The present study investigated the preventive effects of the aqueous extract of the mixture of the aerial parts of *B*. *pilosa* and *C*. *citratus* on a Wistar rat model of hypertension induced by oral administration of alcohol + salt solution at doses of 5 g/kg and 2.46 g/kg, respectively, for 49 days.

Alcohol and salt are the most important causes for HBP and increased cardiovascular risk worldwide [30]. Chronic alcohol and salt abuse is the cause of cardiovascular, renal, and liver dysfunction as well as oxidative stress and dyslipidemias associated with atherosclerosis that contributes to the pathogenesis of hypertension. In the present study, seven weeks of alcohol + salt solution (inducer) consumption resulted in an important increase in blood pressure and heart rate. The increase in those hemodynamic parameters by the inducer was significantly prevented by the substances given concomitantly with the inducer. Dyslipidemias and atherosclerosis, which are risk factors for cardiovascular disease [31], were observed in this study by decreased HDL-chol levels and increased TC, TG, VLDL-chol, and LDL-chol levels as well as atherogenic indices (CRR, AC, and AIP). This damage was confirmed by the histology and histomorphometry of the aorta characterised by thickening of the media. This might be the result of the effects of ethanol and/or salt in rats treated with the inducing solution. Indeed, alcohol could act by increasing the bioavailability of free fatty acids (glycerophosphates), reducing the activity of the enzymes such as lipoprotein lipase and triglyceride lipase, and decreasing the oxidation of fats, thus causing the accumulation of lipids in the serum [32]. These results are in agreement with the work of Olaleye et al. [33] who showed that a high-salt diet (8%) for six weeks caused a significant increase in serum TG, LDL-chol, and TC concentrations and a reduction in serum HDL-chol levels. According to these authors, dyslipidemias observed could promote the development of atheromatous plaques in the arteries and consequently HBP. According to Ikewuchi [28], atherogenic indices are strong predictors of heart disease risk, and the risk of developing cardiovascular diseases increases with increasing values of these indices and vice versa. In the pathogenesis of atherosclerosis described by Badimon and Vilahur [31], the increase in plasma LDL-chol leads to an increase in LDL-chol in the arterial wall. Once in arteries, they promote vascular resistance which leads to AHT. Furthermore, an increase in HDL-chol and a reduction in LDL-chol and TC levels could be considered beneficial in the long-term prognosis of cardiovascular disease [31]. Treatment of rats with spironolactone, the aqueous extract of the mixture of *B*. *pilosa* and *C*. *citratus*, and separate extracts of both plants prevented the onset of dyslipidemia and atherosclerosis induced by alcohol + salt solution. These protective effects were more marked with the mixture of both plant extracts, especially at the lowest dose (100 mg/kg). This confirms a lipid-lowering synergism between the two plants. These results are in agreement with those of Ogbonnia et al. [34] who showed that the hydroethanolic extract of the mixture of *Treculia africana* and *Bryophyllum pinnatum* had better hypoglycaemic and hypolipidemic effects than the extracts of isolate *T*. *africana* or *B*. *pinnatum* on streptozotocin-induced diabetic rats. Therefore, the lipid-lowering effects of this mixture could be attributed to the synergistic effects of each of the bioactive compounds present in each plant. These results confirm the beneficial effects of plant mixtures as hypolipidemic agents and consequently as antihypertensive. These lipid-lowering effects could therefore be one of the mechanisms by which the aqueous extract of the mixture exerts its antihypertensive effect.

In addition to dyslipidemias as risk factors for AHT, alcohol and salt caused cardiovascular dysfunctions characterised by an abnormal increase in the heart rate and blood pressure and a decrease in vasodilators such as nitric oxide. According to Wake (2021), alcohol or its metabolic derivatives act on the cardiovascular system or on the blood pressure regulation systems. This is achieved through a decrease in nitric oxide in the vascular endothelium due to inhibition of endothelial nitric oxide synthase [30]. This leads to inflammatory/oxidative damage to the vascular endothelium and therefore to vasoconstriction which increases vascular resistance and leads to HBP [30]. Alcohol may have a direct effect on smooth muscle cells by increasing both vascular sensitivity to vasomotor amines and the entry of calcium into muscle cells, which leads to hypertension via increased peripheral vascular resistance [30]. Alcohol also induces the activation of the sympathetic nervous system via increased hypothalamic corticoliberin, resulting in hypersecretion of catecholamines, cortisol, angiotensin, and aldosterone responsible for HBP [30]. In animal models of salt-sensitive hypertension, the increase in blood pressure after a salt load is characterised by a decrease in nitric oxide production [35]. Chronic salt consumption induces the production of superoxide anions via increased xanthine oxidase, which causes vasoconstriction through the decrease in nitric oxide [36]. In addition, a high-salt diet leads to K^+^ depletion, with a negative impact on blood pressure regulation, promoting cardiovascular diseases. The concomitant treatment with alcohol + salt and the aqueous extract of the mixture of *B*. *pilosa* and *C*. *citratus* or extracts of isolated plants significantly prevented the increase in blood pressure and heart rate and decrease in nitrite levels. The results obtained with the two extracts of the isolated plants confirm the antihypertensive effects of *B*. *pilosa* [16] and *C*. *citratus* [20] already demonstrated. The prevention of AHT was more marked by the concomitant treatment with alcohol + salt and the aqueous extract of the mixture of *B*. *pilosa* and *C*. *citratus*, especially at the dose of 100 mg/kg. This confirms a synergistic effect between the two plants in preventing HBP. Moreover, these results obtained with the aqueous extract of the mixture of *B*. *pilosa* and *C*. *citratus* corroborate those of Dzeufiet et al. [37] who demonstrated the benefits of the aqueous extract of a mixture of plants to prevent ethanol and fructose-induced hypertension in rats. This phenomenon of beneficial interaction with plants is known as a synergy of pharmacodynamic effects [38]. This suggests that in the aqueous extract of the mixture of *B*. *pilosa* and *C*. *citratus*, bioactive compounds present such as the phytochemicals mainly flavonoids and phenolic compounds act synergistically to cause a reduction in cardiac output and/or total peripheral resistance and consequently a decrease in blood pressure [39]. These effects would therefore be one of the mechanisms by which the aqueous extract of the mixture exerts its antihypertensive effect.

High alcohol and salt consumption is associated with oxidative stress and increased free radicals, which play an important role in oxidative alterations of proteins and the pathogenesis of hypertension [40]. Oxidative stress in the present study is characterised by a decreased level of GSH, SOD, and catalase activity with an increase in the MDA level. Glutathione plays an excellent role in protecting cells from free radical damages by directly scavenging reactive oxygen species [41]. It reduces hydrogen peroxide (H_2_O_2_) production and therefore prevents any deleterious effects such as lipid peroxidation, which is characterised by the production of MDA [42]. SOD and catalase are important antioxidant enzymes that contribute to getting rid of superoxide anion (O_2_^−^) and H_2_O_2_, respectively [43]. Alcohol and salt are thought to act synergistically to create oxidative imbalance. Indeed, chronic alcohol consumption could result in the depletion of antioxidant enzymes due to the excessive generation of O_2_^−^ and H_2_O_2_ [44]. Alcohol leads to the production of free radicals which by lipid peroxidation causes cellular damages accompanied by a significant increase in MDA levels [45]. Excessive salt consumption is known to be associated with increased tissue production of reactive oxygen species [36]. According to Bopda et al. [35], long-term stress due to chronic salt intake induces a massive and toxic amount of reactive oxygen species, leading to a decrease in GSH and SOD levels. In fact, salt consumption enhances O_2_^−^ and H_2_O_2_ generation accompanied by increased NADPH oxidase expression and activity with decreased SOD in the vessels and kidney, contributing to the pathogenesis of hypertension [36]. Treatment of rats with the aqueous extract of the mixture of *B*. *pilosa* and *C*. *citratus* as well as the extracts from isolated plants significantly prevented the installation of oxidative stress observed in hypertensive rats. The lower dose (100 mg/kg) was more effective in preventing oxidative imbalance. These results corroborate those of Eshrat and Hussain [46] who obtained better antioxidant effects with the mixture of *Curcuma longa* and *Abroma augusta* as compared to the two plants used separately in streptozotocin-induced diabetes. Thus, it can be suggested that the synergistic interaction of the bioactive compounds contained in each mixture of plants confers better antioxidant properties to the aqueous extract of the mixture of *B*. *pilosa* and *C*. *citratus*. These free radical-scavenging properties of the aqueous extract of the mixture could therefore be one of the mechanisms by which it would exert its antihypertensive activity.

The development of hypertension in the present study has had a negative impact on the functioning of vital organs such as the kidneys. Oxidative stress is the main pathological pathway involved in renal dysfunction [47]. In the present study, oxidative stress-mediated by chronic alcohol and/or salt consumption contributed to the development of nephrotoxicity through increased water intake, urinary excretion volume, serum creatinine, uric acid, and urea concentrations, as well as decreased creatinine clearance. This renal injury was confirmed in this study by histological analyses characterised by leucocytic infiltration and mesangial expansion. Indeed, alcohol acts by decreasing the number of vasopressin neurons in the hypothalamus, leading to a decrease in plasma antidiuretic hormone levels [48]. This could explain the increase in urinary excretion volume in rats treated concomitantly with water and inducing solution. The increase in water intake is thought to be due to the stimulation of the renin-angiotensin system by salt leading to the production of angiotensin II. The latter will act at the level of the hypothalamus inducing a sensation of thirst which increases water consumption, blood volume, and therefore blood pressure [49]. Urea, creatinine, and uric acid are the catabolites of ammonia, creatine, and purine nucleotides released into blood and eliminated by the kidney, respectively. Due to higher sensitivity of the glomerular region to alcohol and/or salt-induced oxidative damage, the result of the inducing solution intake will be a decrease of the filtration rate and clearance of these substances in the rats [29]. Studies have reported that increased plasma urea and creatinine levels are a consequence of impaired renal function leading to acute glomerulonephritis, nephrosclerosis, and tubular necrosis [50]. In the present study, the concentrations of biochemically and physiologically important electrolytes and minerals were measured. Sodium, potassium, calcium, and magnesium levels were decreased in plasma and increased in the urine of hypertensive rats as compared to normotensive controls. Plasma electrolytes maintain intravascular homeostasis with interstitial and intracellular space. Alcohol consumption induces polyuria and severe loss of electrolytes and minerals which would result in plasma hyponatremia, hypokalemia, hypomagnesaemia, and hypocalcemia [51]. Adewale and Ifudu studies reported that alcohol-generated free radicals cause severe membrane damage, resulting in decreased mineral reabsorption at the distal tubules and ascending branches of the loops of Henle [51]. Salt induces oxidative stress at the renal level through the expression of NADPH oxidase which stimulates the production of O_2_^−^ in mesangial cells and ascending limbic cells [30]. Treatment of rats with inducing solution and the aqueous extract of the mixture of *B*. *pilosa* and *C*. *citratus* and separate extracts of these two plants significantly prevented kidney damages in rats and maintained plasma electrolytes and minerals at normal levels. The nephroprotective effects were the best with the aqueous extract of the mixture of *B*. *pilosa* and *C*. *citratus* especially at the lowest dose (100 mg/kg). These results are in agreement with the work who Manzoor et al. [52] showing nephroprotective effects of different doses of aqueous extracts of *Foeniculum vulgare* seeds, *Solanum nigrum* fruits, and their mixture on gentamicin-induced nephrotoxicity in albino rabbits. According to these authors, the aqueous extract of the mixture of *F*. *vulgare* and *S*. *nigrum* had the best effects as compared to the extracts of each individual plant. These therapeutic effects may be due to the synergistic effects of the phytocompounds (polyphenols and flavonoids) present in each of the plants, which are thought to modify membrane fatty acid composition, fluidity, permeability, and electrolyte homeostasis by reducing alcohol and salt-induced oxidative stress through free radical-scavenging activity. The aqueous extract of the mixture would therefore partly exert its antihypertensive effect due to its nephroprotective properties, knowing the important role played by the kidney in blood pressure regulation. The effects of the mixture were not dose-dependent on lowering blood lipid and nephroprotection; this could be due to antagonism. In fact, the extract of the mixture contained many molecules, some of which could be antagonistic. Therefore, at low doses, the concentration of these antagonistic molecules was low, thus offering no hindrance to the hypolipidemic and nephroprotective causative substances. Similar results were reported by Dzeufiet et al. [53] when working on the effect of *Ceiba pentandra* on type 2 diabetes.

In addition to the imbalance of the prooxidant/antioxidant equilibrium in favor of prooxidants, alcohol and salt are known to produce free radicals, which affect the cellular permeability of hepatocytes, subsequently leading to elevated levels of serum biochemical parameters such as ALT and AST [54]. This marks an impairment of the structural and functional integrity of liver cell membranes, as these cytosolic enzymes are mainly released into circulation after liver cell injury [55]. An increase in ALT enzyme activity is almost always due to hepatocellular injury and is usually accompanied by an increase in AST [55]. In the present study, hypertensive rats showed an increase in serum ALT and AST activities and bilirubin concentration indicating liver damages. These liver damages were prevented by the aqueous extract of the mixture of *B*. *pilosa* and *C*. *citratus* as well as the extracts of the plants taken individually. The present results suggest that the aqueous extract of the mixture of *B*. *pilosa* and *C*. *citratus* (100 mg/kg) is more effective as compared to the aqueous extracts of isolated *B*. *pilosa* or *C*. *citratus*. These results obtained with the mixture's aqueous extract are in agreement with the work of Tripathi and Trilochana [56] who showed that the ethanolic extract of the mixture of the leaves and flowers of *Bassia latifolia* had better hepatoprotective effects than the separate ethanolic extracts of the leaves and flowers in paracetamol-induced hepatotoxic rats. This hepatoprotective activity could be one of the mechanisms whereby the mixture's aqueous extract exerts its antihypertensive effect.

## 5. Conclusion

The results of the present study showed that the aqueous extract of the mixture of *B*. *pilosa* and *C*. *citratus* (100 and 200 mg/kg) significantly prevented the development of hypertension in rats treated with the alcohol + salt solution. According to the biochemical results, which were supported by histopathological evidences, the aqueous extract of the *B*. *pilosa* and *C*. *citratus* mixture, especially at the lower dose, synergistically protected the cardiovascular system as well as the kidney and liver against adverse effects of the alcohol + salt solution compared to the plants taken separately. This study also suggests that the possible antihypertensive mechanisms of this herbal mixture could be attributed to the vasorelaxant, antioxidant, lipid-lowering, hepato, and nephroprotection activities of its phenolic and flavonoid components.

## Figures and Tables

**Table 1:** Bioactive compounds identified in the aqueous extract of the mixture of *B*. *pilosa* and *C*. *citratus*.

Compounds	Identification
Flavonoids	+
Tannins	−
Alkaloids	+
Quinones	+
Saponins	+
Steroids	+
Triterpenes	+
Cardiac glycosides	+
Phenols	+
Anthocyanins	−

+, present; −, absent.

**Table 2:** Quantitative phytochemical screening (determination of polyphenols and flavonoids).

	Polyphenols (mg CE/g extract)	Flavonoids (mg CE/g extract)
*B*. *pilosa* *+* *C*. *citratus*	922.71 ± 3.22	71.48 ± 3.41

CE, catechin equivalent.

**Table 3:** Diuretic activity of the aqueous extract of the mixture of *B*. *pilosa* and *C*. *citratu ****s***.

Parameters	TN	FURO 10	BC 100	BC 200	B 200	C 200
Urinary excretion (%)	259.64	327.07	255.89	359.20	356.86	419.63
Diuretic activity	—	1.00	0.79	1.06	0.96	1.12
Diuretic action	1.00	1.34	1.05	1.43	1.29	1.51

Each value represents the mean (*n* = 6). TN, rats receiving distilled water (10 mL/kg); BC 100 and BC 200, rats receiving the aqueous extract of the mixture of *B*. *pilosa* and *C*. *citratus* at the doses of 100 and 200 mg/kg; FURO 10, rats receiving furosemide at the dose of 10 mg/kg; B 200, rats receiving *B*. *pilosa* aqueous extract at the dose of 200 mg/kg; C 200, rats receiving *C*. *citratus* aqueous extract at the dose of 200 mg/kg.

**Table 4:** Effects of the aqueous extract of the mixture of *B. pilosa* and *C. citratus* on the lipid profile.

Parameters	DW	DW + Al-salt	SPIRO + Al-salt	BC 100 ± Al-salt	BC 200 ± Al-salt	B 200 ± Al-salt	C 200 ± Al-salt
TC (mg/dL)	66.20 ± 1.02	94.27 ± 4.20^3^	82.64 ± 0.222^b^	67.88 ± 2.22^c^	79.17 ± 4.26^1a^	88.06 ± 3.88^3^	92.75 ± 0.20^3^
TG (mg/dL)	27.80 ± 1.95	60.39 ± 5.96^3^	28.15 ± 3.88^c^	22.76 ± 1.87^c^	40.17 ± 3.36^b^	23.68 ± 1.21^c^	27.89 ± 3.92^c^
HDL-chol (mg/dL)	57.07 ± 2.18	47.81 ± 4.30	56.87 ± 1.02	48.80 ± 1.78	63.69 ± 2.50^b^	49.56 ± 1.72	71.92 ± 3.93^c2^
LDL-chol (mg/dL)	3.57 ± 0.81	34.38 ± 4.02^3^	20.14 ± 1.62^3^a	14.53 ± 1.76^3b^	7.45 ± 1.58^1c^	33.76 ± 0.98^3^	15.24 ± 1.03^3b^
VLDL-chol (mg/dL)	5.56 ± 0.39	12.08 ± 1.19^3^	5.63 ± 0.78^c^	4.55 ± 0.37^c^	8.03 ± 0.67^b^	4.74 ± 0.24^c^	5.58 ± 0.78^c^

Each value represents the mean ± SEM (*n* = 6). TC, total cholesterol; LDL-chol, low-density lipoprotein cholesterol; HDL-chol, high-density lipoprotein cholesterol; TG, triglycerides; VLDL-chol, very-low-density lipoprotein cholesterol; DW, normal rats receiving distilled water only (10 mL/kg); DW + Al-salt, rats receiving distilled water (10 mL/kg) and the inducer (alcohol + salt solution) simultaneously; BC 100+Al-salt and BC 200+Al-salt, rats receiving the aqueous extract of the mixture of *B*. *pilosa* and *C*. *citratus*, respectively, at doses of 100 and 200 mg/kg and the inducer; SPIRO + Al-salt, rats receiving spironolactone (10 mg/kg) and the inducer simultaneously; B 200+Al-salt, rats receiving *B*. *pilosa* aqueous extract (200 mg/kg) and the inducer simultaneously; C 200+Al-salt, rats receiving concurrent *C*. *citratus* aqueous extract (200 mg/kg) and the inducer; ^1^*p* < 0.05; ^2^*p* < 0.01; 3 *p* < 0.001, significant difference compared to normal rats receiving distilled water only; ^a^*p* < 0.05; ^*b*^*p* < 0.01; ^c^*p* < 0.001, significant difference compared to rats receiving distilled water and the inducer.

**Table 5:** Effects of the aqueous extract of the mixture of *B*. *pilosa* and *C*. *citratus* on atherogenic indices.

Atherogenic indices	DW	DW + Al-salt	SPIRO + Al-salt	BC 100 + Al-salt	BC 200 + Al-salt	B 200 + Al-salt	C 200 + Al-salt
CRR	1.16 ± 0.08	1.97 + 0.33^3^	1.46 ± 0.03^c^	1.40 ± 0.06^c^	1.25 ± 0.09^c^	1.80 + 0.13^3^	1.31 ± 0.08^c^
AC	0.16 ± 0.03	0.97 + 0.07^3^	0.45 ± 0.03^a1^	0.39 ± 0.06^b^	0.24 ± 0.03^c^	0.78 + 0.08^3^	0.29 ± 0.08^c^
AIP	−0.31 ± 0.03	0.20 + 0.04^3^	−0.32 ± 0.06^c^	−0.34 ± 0.03^c^	−0.21 ± 0.05^c^	−0.32 ± 0.01^c^	−0.43 ± 0.08^c^

Each value represents the mean ± SEM (*n* = 6). CRR, cardiac risk ratio; AC, atherogenic coefficients; AIP, atherogenic index of plasma; DW, normal rats receiving only distilled water (10 mL/kg); DW + Al-salt, rats receiving distilled water (10 mL/kg) and the inducer (alcohol + salt solution) simultaneously; BC 100+Al-salt and BC 200+Al-salt, rats receiving the aqueous extract of the *B*. *pilosa* and *C*. *citratus* mixture at doses of 100 and 200 mg/kg, respectively, and the inducer; SPIRO + Al-salt, rats receiving spironolactone (10 mg/kg) and the inducer simultaneously; B 200+Al-salt, rats receiving *B*. *pilosa* aqueous extract (200 mg/kg) and the inducer; C 200 + Al-salt, rats receiving *C*. *citratus* aqueous extract (200 mg/kg) and the inducer simultaneously; ^1^*p* < 0.05; ^3^*p* < 0.001, significant difference compared to normal rats receiving only distilled water; ^a^*p* < 0.05; ^*b*^*p* < 0.01; ^c^*p* < 0.001, significant difference compared to rats receiving distilled water and the inducer.

**Table 6:** Effects of the aqueous extract of the mixture of *B*. *pilosa* and *C*. *citratus* on liver function.

Parameters	DW	DW + Al-salt	SPIRO + Al-salt	BC 100 ± Al-salt	BC 200+Al-salt	B 200 ± Al-salt	C 200 ± Al-salt
ALT (U/L)	12.36 ± 6.46	92.20 ± 7.87^3^	38.41 ± 2.29^c^	40.74 ± 2.02^c^	21.73 ± 1.25^c3^	35.70 ± 1.16^c^	67.90 ± 1.74^c3^
AST (U/L)	67.73 ± 3.78	95.10 ± 6.95^3^	67.96 ± 6.76^c^	59.49 ± 6.48^c^	80.49 ± 8.12^b^	70.16 ± 4.64^c^	67.90 ± 5.04^c^
Albumin (mg/dL)	3.67 ± 0.25	2.76 ± 0.16^2^	3.14 ± 0.08	3.37 ± 0.18	3.32 ± 0.11	3.15 ± 0.04	3.36 ± 0.14
Total bilirubin (mg/dL)	0.18 ± 0.01	0.24 ± 0.01^2^	0.19 ± 0.01^a^	0.13 ± 0.01^c^	0.18 ± 0.01^b^	0.19 ± 0.01^a^	0.17 ± 0.01^b^

Each value represents the mean ± SEM (*n* = 6). DW, normal rats receiving only distilled water (10 mL/kg); DW + Al-salt, rats receiving distilled water (10 mL/kg) and the inducer (alcohol + salt solution) simultaneously; BC 100 + Al-salt and BC 200 + Al-salt, rats receiving the aqueous extract of the mixture of *B*. *pilosa* and *C*. *citratus* at doses of 100 and 200 mg/kg, respectively, and the inducer; SPIRO + Al-salt, rats receiving spironolactone (10 mg/kg) and the inducer simultaneously; B 200 + Al-salt, rats receiving *B*. *pilosa* aqueous extract (200 mg/kg) and the inducer; C 200 + Al-salt, rats receiving the aqueous extract of *C*. *citratus* (200 mg/kg) and the inducer; ^1^*p* < 0.05; ^2^*p* < 0.01; ^3^*p* < 0.001, significant difference compared to normal rats receiving only distilled water; ^a^*p* < 0.05; ^*b*^*p* < 0.01; ^c^*p* < 0.001, significant difference compared to rats receiving distilled water and the inducer.

**Table 7:** Effects of the aqueous extract of the mixture of *B*. *pilosa* and *C*. *citratus* on markers of renal function at the serum level.

Parameters	DW	DW + Al-salt	SPIRO + Al-salt	BC 100 + Al-salt	BC 200 + Al-salt	B 200 + Al-salt	C 200 + Al-salt
Creatinine (mg/dL)	0.77 ± 0.02	1.06 + 0.01^3^	0.74 ± 0.01^c^	0.69 ± 0.04^c^	0.86 ± 0.04^b2^	0.63 ± 0.05^c^	0.76 ± 0.01^c^
Creatinine clearance (mL/min)	0.36 ± 0.04	0.18 + 0.02^2^	0.30 ± 0.04	0.43 ± 0.04^c^	0.31 ± 0.02	0.35 ± 0.01^b^	0.28 ± 0.01
Urea (mg/dL)	19.16 ± 1.82	54.30 + 3.01^3^	18.41 ± 2.75^c^	8.00 ± 1.82^1c^	16.33 ± 2.42^c^	21.88 ± 2.39^c^	17.25 ± 2.46^c^
Uric acid (mg/dL)	3.07 ± 0.40	9.28 + 1.06^3^	3.27 ± 0.25^c^	1.74 ± 0.23^c^	3.36 ± 0.17^c^	2.26 ± 0.27^c^	1.97 ± 0.29^c^
Mg^2+^ (mmol/L)	3.63 ± 0.16	2.20 + 0.18^3^	2.67 + 0.09^1^	3.28 ± 0.18^b^	2.62 + 0.13^2^	3.35 ± 0.26^b^	2.92 ± 0.18
Ca^2+^ (mmol/L)	1.43 ± 0.03	1.22 ± 0.21	1.21 ± 0.01	1.25 ± 0.01	1.19 ± 0.01	1.61 ± 0.01	0.85 ± 0.02
Na^+^(mmol/L)	125.90 ± 1.65	103.09 + 3.42^3^	124.90 ± 3.82^c^	130.30 ± 0.04^c^	119.20 ± 1.45^b^	109.60 + 2.72^2^	132.30 ± 2.84^c^
K^+^(mmol/L)	9.11 ± 1.04	4.88 + 0.27^3^	6.85 ± 0.72	6.71 ± 0.33	6.66 ± 0.08	7.41 ± 0.96	5.29±0.23^2^

Each value represents the mean ± SEM (*n* = 6). DW, normal rats receiving only distilled water (10 mL/kg); DW + Al-salt, rats receiving distilled water (10 mL/kg) and the inducer (alcohol + salt solution) simultaneously; BC 100 + Al-salt and BC 200+Al-salt, rats receiving the aqueous extract of the mixture of *B*. *pilosa* and *C*. *citratus*, respectively, at doses of 100 and 200 mg/kg and the inducer; SPIRO + Al-salt, rats receiving spironolactone (10 mg/kg) and the inducer simultaneously; B 200+Al-salt, rats receiving *B*. *pilosa* aqueous extract (200 mg/kg) and the inducer; C 200+Al-salt, rats receiving *C*. *citratus* aqueous extract (200 mg/kg) and the inducer simultaneously. ^1^*p* < 0.05; ^2^*p* < 0.01; ^3^*p* < 0.001, significant difference compared to normal rats receiving distilled water only; ^*b*^*p* < 0.01; ^*c*^*p* < 0.001, significant difference compared to rats receiving distilled water and the inducer.

**Table 8:** Effects of the aqueous extract of the mixture of *B*. *pilosa* and *C*. *citratus* on markers of renal function at the urinary level.

Parameter	DW	DW + Al-salt	SPIRO + Al-salt	BC 100 + Al-salt	BC 200+Al-salt	B 200 + Al-salt	C 200 + Al-salt	
Creatinine (mg/dL)	23.27 ± 1.52	11.43 + 0.44^3^	14.29 ± 1.16	20.68 ± 0.71^c^	16.33 ± 0.34^b^	14.42 ± 0.66	14.83 ± 0.14	
Urea x 100 (mg/dL)	20.22 ± 4.89	4.11 + 1.27^3^	10.33 ± 2.46	41.33 ± 4.94^2c^	23.89 ± 3.38^a^	28.78 ± 4.82^b^	19.92 ± 3.15^a^	
Uric acid (mg/dL)	20.10 ± 1.43	8.90 + 0.36^3^	15.42 ± 1.57^1c^	9.89 + 0.50^3^	9.89 + 0.25^3^	8.28 + 0.36^3^	13.33 ± 0.64^3a^	
Ca^2+^. (mmol/L)	3.54 ± 0.40	3.42 ± 0.40	4.92 ± 0.28	4.10 ± 0.60	4.12 ± 0.36	4.40 ± 0.38	4.43 ± 0.09	
Mg^2+.^ (mg/dL)	5.83 ± 1.24	15.05 + 0.83^3^	10.47 ± 1.78	11.23 ± 1.08	9.95 ± 1.25	14.30 + 0.41^2^	14.81 + 1.18^2^	
K^+^ (mmol/L)	26.46 ± 2.38	74.56 + 2.48^3^	35.12 ± 2.82^c^	24.85 ± 1.51^c^	37.90 ± 2.22^c^	36.34 ± 2.92^c^	43.48 ± 2.49^c^	
Na^+.^ (mmol/L)	317.50 ± 11.41	422.50 + 23.26^3^	318.80 ± 19.26^c^	290.50 ± 3.53^c^	291.00 ± 6.99^c^	318.50 ± 4.78^c^	401.30 ± 3.65^c^	

Each value represents the mean ± SEM (*n* = 6). DW, normal rats receiving only distilled water (10 mL/kg); DW + Al-salt, rats receiving distilled water (10 mL/kg) and the inducer (alcohol + salt solution) simultaneously; BC 100+Al-salt and BC 200+Al-salt, rats receiving the aqueous extract of the mixture of *B*. *pilosa* and *C*. *citratus*, respectively, at doses of 100 and 200 mg/kg and the inducer; SPIRO + Al-salt, rats receiving spironolactone (10 mg/kg) and the inducer simultaneously; B 200+Al-salt, rats receiving *B*. *pilosa* aqueous extract (200 mg/kg) and the inducer; C 200+Al-salt, rats receiving *C*. *citratus* aqueous extract (200 mg/kg) and the inducer simultaneously. ^2^*p* < 0.01; ^3^*p* < 0.001, significant difference compared to normal rats receiving distilled water only; ^a^*p* < 0.05; ^*b*^*p* < 0.01; ^*c*^*p* < 0.001, significant difference compared to hypertensive rats.

## Data Availability

The data used to support the findings of this study are available from the corresponding author upon request.
